# Development and evaluation of a text analytics algorithm for automated application of national COVID-19 shielding criteria in rheumatology patients

**DOI:** 10.1136/ard-2024-225544

**Published:** 2024-04-04

**Authors:** Meghna Jani, Ghada Alfattni, Maksim Belousov, Lynn Laidlaw, Yuanyuan Zhang, Michael Cheng, Karim Webb, Robyn Hamilton, Andrew S Kanter, William G Dixon, Goran Nenadic

**Affiliations:** 1 Centre for Epidemiology Versus Arthritis, Centre for Musculoskeletal Research, The University of Manchester, Manchester, UK; 2 Department of Rheumatology, Northern Care Alliance NHS Foundation Trust Salford Care Organisation, Salford, UK; 3 NIHR Manchester Biomedical Research Centre, Manchester University NHS Foundation Trust, Manchester Academic Health Science Centre, Manchester, UK; 4 Department of Computer Science, The University of Manchester, Manchester, UK; 5 Department of Computer Science, Jamoum University College, Umm Al-Qura University, Makkah, Saudi Arabia; 6 Department of Business Intelligence, Northern Care Alliance NHS Foundation Trust, Salford Care Organisation, Salford, UK; 7 Department of Biomedical Informatics, Columbia University, New York, New York, USA

**Keywords:** Covid-19, Biological Therapy, Epidemiology

## Abstract

**Introduction:**

At the beginning of the COVID-19 pandemic, the UK’s Scientific Committee issued extreme social distancing measures, termed ‘shielding’, aimed at a subpopulation deemed extremely clinically vulnerable to infection. National guidance for risk stratification was based on patients’ age, comorbidities and immunosuppressive therapies, including biologics that are not captured in primary care records. This process required considerable clinician time to manually review outpatient letters. Our aim was to develop and evaluate an automated shielding algorithm by text-mining outpatient letter diagnoses and medications, reducing the need for future manual review.

**Methods:**

Rheumatology outpatient letters from a large UK foundation trust were retrieved. Free-text diagnoses were processed using Intelligent Medical Objects software (Concept Tagger), which used interface terminology for each condition mapped to Systematized Medical Nomenclature for Medicine–Clinical Terminology (SNOMED-CT) codes. We developed the Medication Concept Recognition tool (Named Entity Recognition) to retrieve medications’ type, dose, duration and status (active/past) at the time of the letter. Age, diagnosis and medication variables were then combined to calculate a shielding score based on the most recent letter. The algorithm’s performance was evaluated using clinical review as the gold standard. The time taken to deploy the developed algorithm on a larger patient subset was measured.

**Results:**

In total, 5942 free-text diagnoses were extracted and mapped to SNOMED-CT, with 13 665 free-text medications (n=803 patients). The automated algorithm demonstrated a sensitivity of 80% (95% CI: 75%, 85%) and specificity of 92% (95% CI: 90%, 94%). Positive likelihood ratio was 10 (95% CI: 8, 14), negative likelihood ratio was 0.21 (95% CI: 0.16, 0.28) and F1 score was 0.81. Evaluation of mismatches revealed that the algorithm performed correctly against the gold standard in most cases. The developed algorithm was then deployed on records from an additional 15 865 patients, which took 18 hours for data extraction and 1 hour to deploy.

**Discussion:**

An automated algorithm for risk stratification has several advantages including reducing clinician time for manual review to allow more time for direct care, improving efficiency and increasing transparency in individual patient communication. It has the potential to be adapted for future public health initiatives that require prompt automated review of hospital outpatient letters.

**WHAT IS ALREADY KNOWN ON THIS TOPIC**

- In April 2020, several Western countries used varying population-level strategies to identify patients who were clinically extremely vulnerable (CEV) to help mitigate the most severe adverse outcomes associated with COVID-19.
- For rheumatology patients in the UK, identification of these subgroups was based on a national risk-based score incorporating age, comorbidities and being on immunosuppressive therapies including biologics. It required manual review of semistructured clinical letters by rheumatology teams at a time, which was resource-intensive and inefficient.
- Natural language processing, a branch of artificial intelligence, offers opportunities to extract meaningful information from unstructured text data; however, there are few large-scale implemented examples in rheumatology.

**WHAT THIS STUDY ADDS**

- A text analytics-based algorithm for automated application of the COVID-19 CEV criteria was developed and applied to rheumatology clinical letters.
- Compared with manual scores performed by clinicians during the pandemic, it performed well with a sensitivity of 80% and specificity of 92%, with deployment of the algorithm requiring considerably less time.

**HOW THIS STUDY MIGHT AFFECT RESEARCH, PRACTICE OR POLICY**

- An automated algorithm for risk stratification could help reduce clinician time for manual review to allow more time for direct care and rapid deployment of a complex set of rules on all patient records, improving efficiency and transparently communicating decisions based on individual patient risk.
- It could also be adapted for future clinical use requiring rapid risk stratification of patients for pandemic planning or in other public health initiatives.

## Introduction

In April 2020, at the start of the COVID-19 pandemic, the UK’s Scientific Committee issued extreme social distancing measures termed ‘shielding’. These were aimed at a subset of the UK population who were deemed clinically extremely vulnerable (CEV) to infection.^1^ Approximately 4.1 million patients over the course of the pandemic who were identified as CEV and perceived at increased risk of becoming seriously ill from COVID-19 were therefore asked to shield.^2 3^ It was advised that these individuals should not leave their homes and avoid face to-face contact based on individual risk factors.

**Figure 1:**
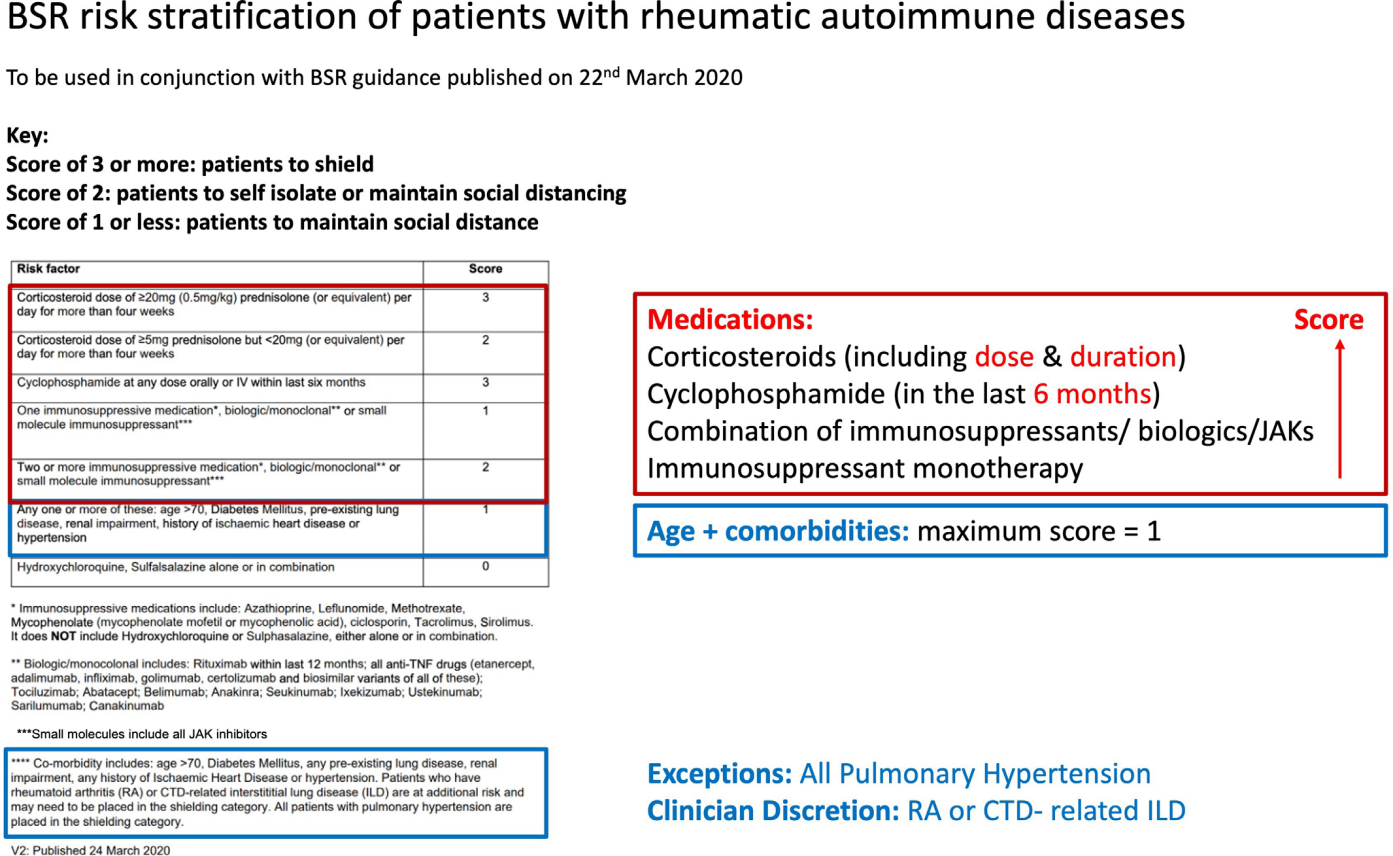
British Society for Rheumatology (BSR) risk stratification score. Diagnoses-related scoring is indicated in blue and medication-related scoring is indicated in red. The BSR score was heavily weighted towards medication use, with the requirement of dose and duration for steroids and temporal information required for cyclophosphamide. Comorbidities and age scored a maximum of 1 point. The exceptions were those with pulmonary hypertension, and those with rheumatoid arthritis or connective tissue disease-associated ILD requiring clinical discretion. CTD, connective tissue disease; ILD, interstitial lung disease; IV, intravenously; JAK, Janus kinase; TNF, tumour necrosis factor.

To guide efficient and systematic identification of these patients, national specialty societies produced risk stratification tools based on a patient’s age, immunosuppressive therapies and diagnoses directed at clinicians to quickly identify such patients (figure 1). In outpatient-based specialties, such as rheumatology, where communication is commonly based on unstructured outpatient clinic letters, it was not possible to run a rapid search for patients with diagnostic and medication codes. Diagnoses for outpatient visits, outpatient prescribed medications (ie, majority of high-cost drugs such as biologics) and measures such as disease severity are only recorded in semistructured letters and are thus not machine readable, searchable or analysable across the population. Hospital clinicians needed to manually review sequential clinic letters of patients to manually score their risk as per national guidance and identify which patients should receive communications about shielding measures. This took whole rheumatology teams many dedicated sessions amounting to scores of hours to read through recent letters for all their patient cohort.

Given the considerable clinician time required, manual evaluation of clinical records can be inefficient and inconsistent. This approach may not allow evaluation of every single patient attending the department due to the large patient volume. Reviewing only a subset of patients based on pre-existing likelihood of being high risk is error-prone and would likely miss many patients, and manually reviewing every patient record is cost and time prohibitive. A recent retrospective analysis based on National Health Service (NHS) digital data shows substantial regional variation in the proportions of people who were asked to shield across the country.^2 4^ While these differences may be in part due to variation in the prevalence of long-term conditions, the heterogeneity in the approaches between clinical teams due to workload pressures to identify CEV patients is likely to contribute to these discrepancies.^4^ In hospitals where manual evaluation was not possible due to clinical workload pressures, strategies included sending a generic letter to all patients about shielding and asking patients to self-identify if they met the set of complex criteria. However, personal interpretation of the complex guidance may lead to added anxiety during a period that was fraught with uncertainty. This approach also put the onus on the patient informing clinical teams about their CEV status to access additional support. For example, timely shielding classification was especially important to CEV patients for practical reasons such as obtaining a supermarket priority delivery slot and to provide evidence for the need to work from home.^5^ The process of identifying individuals to go onto the shielded list of patients was retrospectively critiqued for being reliant on inaccessible data and a ‘lack of joined up systems’.^6^

In this retrospective study, we explored the feasibility and potential benefits of using an automated approach to assist in efficient identification of patients who needed shielding, with a future benefit of adaptability as the guidance evolved. We based our approach on automated text analytics, which we applied to rheumatology department outpatient letters in a large foundation trust hospital providing secondary and tertiary-level care. Our aim was to implement and evaluate an algorithm that used patient demographics, automatically text-mined diagnoses and medications from outpatient letters to apply shielding rules in rheumatology. If successful, this could reduce the need for manual review of such tasks in the future. Specific objectives were to:

1. Automatically extract and code comorbidities and medication exposures from outpatient letters.
2. Implement and assess the performance of an algorithm to apply the British Society for Rheumatology (BSR) shielding guidelines, comparing identification of shielding requirements in a subset of patients with an automated approach with the manual record review by clinicians.
3. Evaluate the time required to deploy the automated algorithm on electronic health records (EHRs) of all patients who attended the rheumatology clinic with at least two clinical letters prior to April 2020, when the shielding guidance was issued.

## Methods

### Data source

We retrieved outpatient letters for this study from the rheumatology department of Salford Royal Hospital, part of the Northern Care Alliance, a large foundation trust in the UK. Outpatient letters in the UK are the main method of communication between hospitals and primary care physicians. They are written as per the Professional Records Standards Body (PRSB)^7^ guidance and comprise of semistructured data that include relevant diagnoses and medications (online supplemental figure 1) with additional free-text narrative. Since publication of the PRSB guidelines, it is expected that all outpatient clinical letters would follow this standard format in the UK.

10.1136/ard-2024-225544.supp1 Supplementary data

### Extraction of diagnoses and medications

We retrieved all letters between 10 June 2013, when semistructured letters were consistently implemented in the hospital according to the national PRSB published guidelines, and 13 November 2020. Prior to June 2013, there was considerable heterogeneity in how clinicians structured their own outpatient letters. To obtain an appropriate level of clinical data required to address the objectives, we retrieved the two most recent letters prior to 1 April 2020 for each patient, with information about diagnoses and medications taken from the semistructured part of the letter only. We extracted outpatient letter subheading titles from the semistructured part of letters initially (n=3465) and reviewed by the research team. Headings used to denote diagnoses (eg, ‘rheumatological diagnoses’, ‘other diagnoses’) and medications (eg, ‘current medications’, ‘previous DMARDs’ (disease-modifying antirheumatic drugs)) were then selected. The hospital data science team used these headings to extract the relevant lists of diagnosis and medication text from the semistructured part of the letters to run the subsequent algorithms. No personal identifiable information was available to the research team.

**Figure 2:**
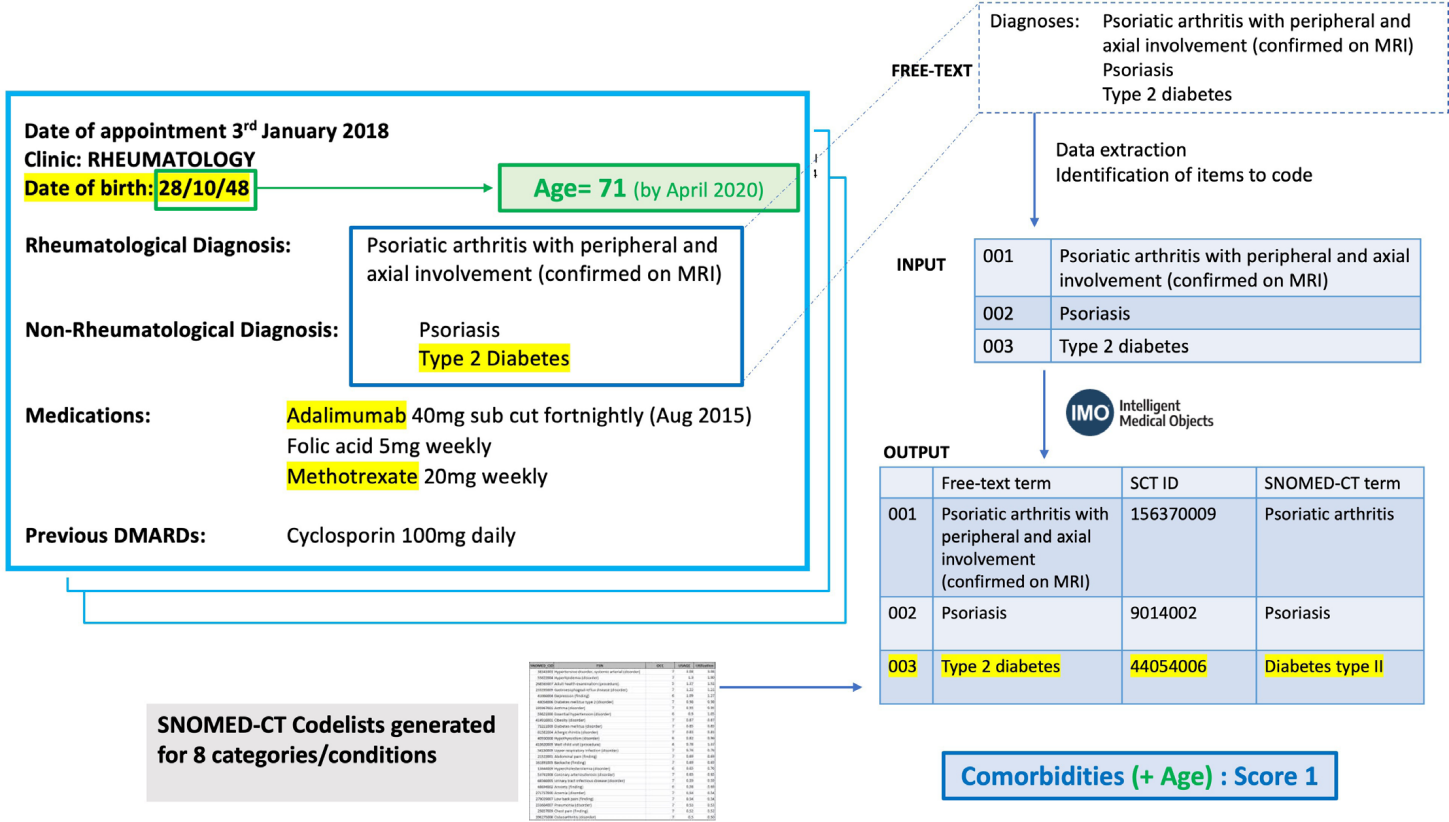
Worked example of how age and diagnoses from letters were combined to contribute to the patient shielding status. The highlighted areas of the semistructured letter contributed to the shielding status in this example.

The lists of diagnoses were processed with Intelligent Medical Objects (IMO) software^8^ (Concept Tagger), which used IMO’s core interface terminology to map each free-text description of a condition to Systematized Nomenclature of Medicine Clinical Terms (SNOMED-CT) codes. IMO was chosen as the software as it specialises in developing, managing and licensing medical vocabularies, and it had an existing partnership in place with the research team to evaluate its performance compared with manual coding performed by experienced clinicians. In our previous work, we had demonstrated that the performance of this software is comparable with human coding of diagnoses.^9^ SNOMED-CT codes were then mapped to lists of codes for each specific comorbidity (figure 2) that was used in the risk scoring according to the BSR shielding guidance figure 1. For the purposes of this work, we assumed all diagnoses in the letter be current diagnoses.

**Figure 3:**
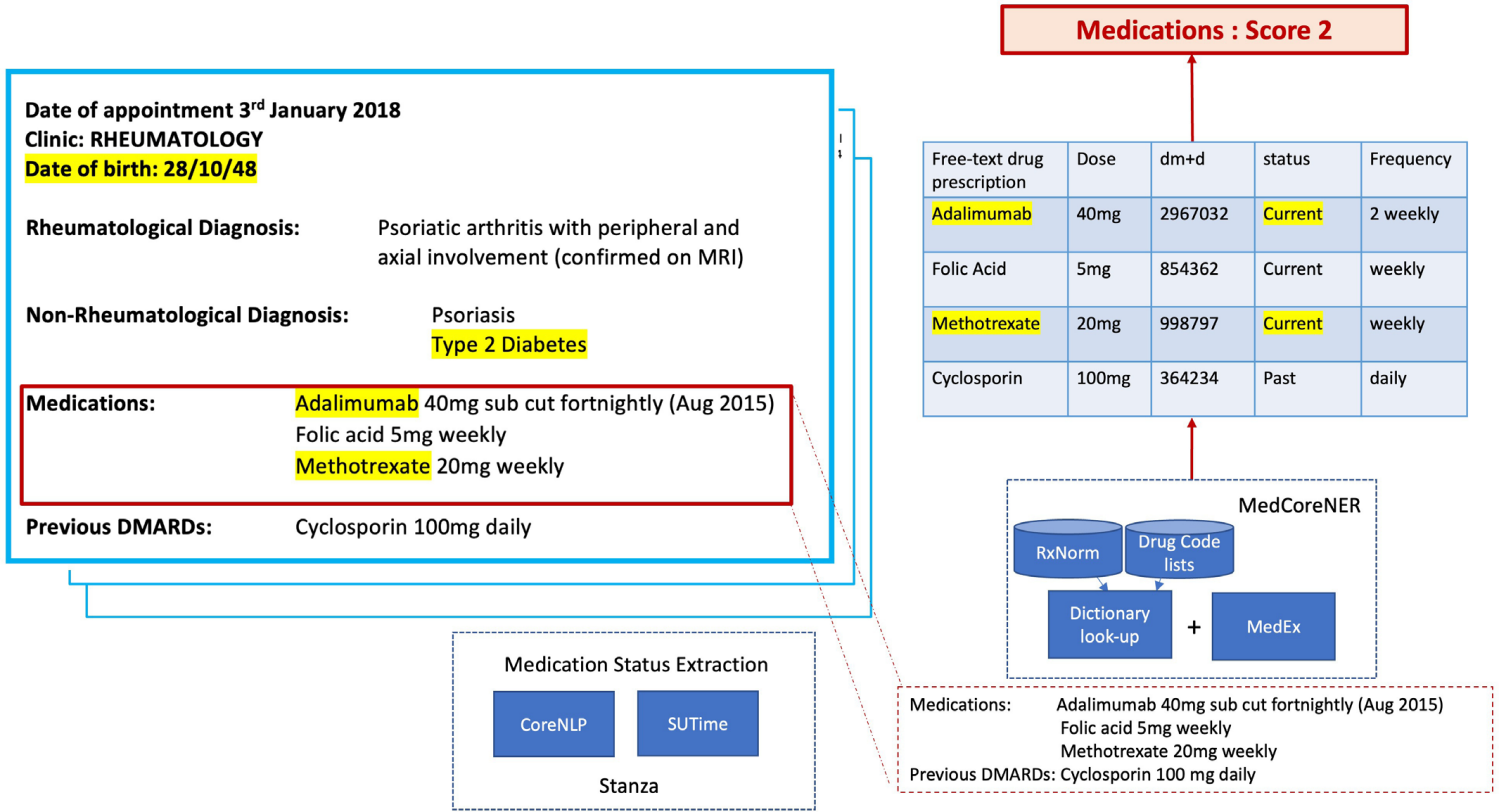
Worked example of how medications from letters were combined to contribute to the patient shielding status. The highlighted areas of the semistructured letter contributed to the shielding status in this example. The figure shows the specific software used for medication extraction and text-mining. Stanza is a python natural language processing package for linguistic analysis consists of a collection of tools including a library for processing temporal expressions such as ‘May 4th, 2019’.

Based on previous work,^10 11^ we developed the Medication Concept Recognition tool (Named Entity Recognition) to retrieve medications (including their type, for example, immunosuppressant), dose, duration and the status (active/past) at the time of the letter from their textual description (figure 3). We used a combination of existing text-mining tools, including MedEx and Stanza, to process the data and extract the necessary information (figure 3). The medication status: past, current/active, planned medications or unknown, was established mainly based on the heading where they appeared. For instance, a medication appearing under ‘previous DMARDs’ meant that the medication’s status was past. The extraction algorithm also considered any additional information that might have been reported in the free-text medication list. This included any information that indicated that a medication was stopped or not started, even though it appeared under ‘current medication’. RxNorm relationships were used to map identified medications to the drug classes required for shielding decisions (eg, ‘Humira’ to ‘biological drugs’). Only those medications which were ‘active’ in April 2020 contributed towards scoring by the algorithm.

We consolidated data for each patient by combining information from the latest two letters prior to April 2020, which were assumed to contain the most up-to-date patient information on diagnoses and active medications. For the information that appeared in both letters such as data on a patient has a particular comorbidity or on a particular medication, we kept only the information from the most recent letter, along with the date of the clinical encounter. If the diagnosis and medication information from the last two letters was different, the latest letter’s information provided the input for the algorithm, as would be commonly observed with medication/dose changes.

### Shielding algorithm

The BSR published a rule-based score that specified numerical thresholds for three categories: *shielding* (score of 3 or above), *self-isolation/social distancing* according to patient discretion (score of 2) or *social distancing* alone as per the current government guidance (score of 1 or 0). Figure 1 illustrates the weight provided to different diagnoses and medications.

The rule as specified by the BSR scoring grid was heavily weighted on specific medications such as biological drugs, immunosuppressive medications, glucocorticoids and cyclophosphamide. Highest scores were allocated to higher doses and recent exposure to glucocorticoids or being on cyclophosphamide within the last 6 months, requiring text-mining of dose and timing of these medications (figure 1). Being over 70 years old or having one of several comorbidities added an additional 1 point. The exception was patients with pulmonary hypertension, who were recommended to be automatically placed in the shielding category. For instance, a patient aged 71 years on 1 April 2020 with type 2 diabetes (1 point total), on adalimumab and methotrexate (2 points) would score a total of 3 points, therefore requiring them to shield (figure 3). Following dissemination of the BSR scoring grid, a similar risk stratification guide (without scores) was also published including measures such as disease activity alongside medication use (online supplemental figure 2). For the purposes of developing the algorithm, we focused on the BSR scoring grid as the default input, as it was easier to interpret clinically and was the one most implemented by the hospital clinical team. The medication, diagnosis and demographic variables were combined to output a score computed by an algorithm with R (V.4.2.2).

*Comparison with gold standard*. At the time of the pandemic, manual review by the rheumatology consultant team was performed in a subset of rheumatology patients deemed likely to fall into the CEV category. Patient records for manual review were identified from pharmacy records indicating prescription of high-cost drugs (eg, biologics and Janus kinase inhibitors), cyclophosphamide and specific scoring conventional synthetic DMARDs (eg, methotrexate), as groups most likely to require shielding based on the BSR criteria. In the absence of being able to easily search for rheumatological diagnoses or medications as this information was not machine readable, locally held databases with details for patients with myositis and systemic sclerosis were also reviewed, as the hospital is a tertiary referral site for these conditions. For the purposes of this study, these decisions were deemed as the ‘gold standard’ against which the performance of the algorithm was calculated using standard performance metrics including sensitivity, specificity and F1 score. F1 score is a measure of predictive performance with values ≥0.7 indicating a good score. Statistical Disclosure Control was applied in line with the ethics approvals for the project.

### Evaluation

Following algorithm development, all algorithm mismatches were manually reviewed by a clinical researcher on the binary measure of shielding or not. We first calculated the number of mismatched patients who had been identified as requiring shielding as per the algorithm but not through manual review (false positives), and vice versa (false negatives). These cases were then reviewed by evaluating the semistructured pseudonymised data (age, diagnoses, medications) extracted from letters without personal identifiable information, rather than the source letters themselves in line with the study approvals.

*Application of the algorithm to all patients and processing time.* Following development of the shielding algorithm and iterative improvements on the data where a gold standard was available, we deployed the automated algorithm on a separate set of EHRs. This included all patients who attended the rheumatology department with at least two clinical letters prior to April 2020, excluding those from the training dataset described above. The time required to deploy the algorithm, once developed, was recorded.

### Patient and public involvement

This work was informed and refined following discussions between a COVID-19 working group that included three to four patient representatives with long-term musculoskeletal conditions, one of whom had experienced a hospital admission with COVID-19. One patient partner who was interested in this work collaborated with the research and NHS teams on this project (LL) and contributed to the protocol and manuscript.

## Results

**Figure 4:**
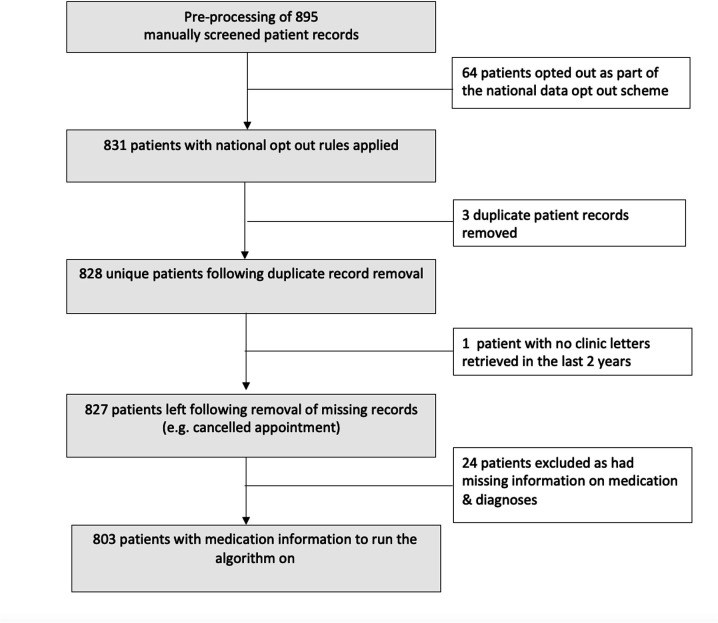
Cohort derivation for the study.

To train the algorithm and compare with manual decisions, we focused on 895 patients who were reviewed clinically at the start of the pandemic. Of 895 patients, 64 patients (7.1%) had not consented for their data to be used for research as part of the national opt-out scheme, hence have been excluded from subsequent analysis. Following removal of duplicate patients (n=3) and missing records (n=24), 803 patient records were available for analysis. Cohort derivation to obtain the final training data is described in figure 4.

In this cohort, the baseline characteristics of the patients identified as those requiring shielding (29.8%, n=239) compared with those who did not (70.2%, n=564) following algorithm deployment, are presented in table 1. Patients in the shielding category were older (mean age 67 years, SD 13) compared with those who were identified as not requiring shielding (55 years; SD 14). Patients identified as requiring shielding had a higher proportion of specific comorbidities such as pre-existing lung disease (n=123; 51%), diabetes (n=49; 21%) and rheumatoid arthritis (n=164; 69%), as well as being on glucocorticoids (n=84; 35%), in line with the BSR guidance (table 1).

**Table 1:** Baseline characteristics of the cohort

	All patients (n=803)	Patients identified as requiring shielding (n=239)	Patients identified as not requiring shielding (n=564)
Age group (years), n (%)			
Mean (SD)	58 (14)	67 (13)	55 (14)
18–39	98 (12)	8 (4)	90 (16)
40–54	215 (27)	37 (15)	178 (31)
55–69	296 (37)	84 (35)	212 (38)
≥70	194 (24)	110 (46)	84 (15)
Female sex, n (%)	480 (60)	158 (66)	322 (57)
Comorbidities, n (%)			
Diabetes (type 1 or type 2)	86 (11)	49 (21)	37 (7)
Ischaemic heart disease	41 (5)	30 (13)	11 (2)
Hypertension	174 (22)	94 (39)	80 (14)
Pre-existing lung disease	209 (26)	123 (51)	86 (15)
Chronic kidney impairment	37 (5)	18 (8)	19 (3)
Pulmonary hypertension	NA	NA	NA
Rheumatoid arthritis	380 (47)	164 (69)	216 (38)
Psoriatic arthritis	174 (22)	27 (11)	147 (26)
Ankylosing spondylitis	136 (17)	21 (9)	115 (20)
Medications, n (%)			
Glucocorticoids (%)	109 (14)	84 (35)	25 (4)
Immunosuppressants (eg, methotrexate, azathioprine)	398 (50)	144 (60)	254 (45)
Biological drugs (eg, tumour necrosis factor inhibitors, rituximab)	419 (52)	132 (55)	287 (51)
Small molecule immunosuppressants (e.g., JAK inhibitors)	NA	NA	NA
Cyclophosphamide	NA	NA	NA

NA=due to small cells/statistical disclosure control to protect patient confidentiality.

JAK, Janus kinase; NA, not available.

A total of 5942 free-text diagnoses were extracted and mapped to SNOMED-CT (average 7 per patient), along with a list of 13 665 free-text medication descriptions (average 17 per patient). This included 11 339 medications (83%) as those identified as being on current/active in the most recent letter prior to April 2020; 2130 medications (16%) as past medications; 164 (1%) as planned medications to be commenced in the future and 32 (0.2%) that were of unknown status.

The automated algorithm for numerical risk scoring (figure 1) performed well and demonstrated a sensitivity of 80% (95% CI: 75%, 85%) and specificity of 92% (95% CI: 90%, 94%). Positive likelihood ratio was 10 (95% CI: 8, 14) and negative likelihood ratio was 0.21 (95% CI: 0.16, 0.28). False positive rate was 8%, while false negative rate was 20% (table 2). The performance of the algorithm when implementing the rules of the BSR stratification guide that incorporated disease activity (online supplemental figure 2) was very similar, with a sensitivity of 80% and a specificity of 92% (table 3).

**Table 2:** Confusion matrix for the BSR scoring grid (numerical)

	True positive	True negative
Predicted positive	192	43
Predictive negative	47	521
Totals	239	563
Sensitivity (95% CI)	80% (75%, 85%)	
Specificity (95% CI)	92% (90%, 94%)	
Positive likelihood ratio (95% CI)	11 (8, 14)	
Negative likelihood ratio (95% CI)	0.21 (0.16, 0.28)	
Positive predictive value (95% CI)	82% (77%, 86%)	
Negative predictive value (95% CI)	92% (90%, 94%)	
False positive rate	7%	
False negative rate	20%	
F1 score	0.81	

BSR, British Society for Rheumatology.

**Table 3:** Confusion matrix for the BSR risk stratification guide (non-numerical)

	True positive	True negative
Predicted positive	192	45
Predictive negative	47	519
Total	239	564
Sensitivity (95% CI)	80% (75%, 85%)	
Specificity (95% CI)	92% (90%, 94%)	
Positive likelihood ratio (95% CI)	10 (8, 14)	
Negative likelihood ratio (95% CI)	0.21 (0.16, 0.28)	
Positive predictive value (95% CI)	81% (76%, 85%)	
Negative predictive value (95% CI)	92% (90%, 94%)	
False positive rate	8%	
False negative rate	20%	
F1 score	0.81	

BSR, British Society for Rheumatology.

### Evaluation of mismatches

Of the 43 patients who were identified as positive when the BSR scoring grid rules were deployed by the algorithm but where the clinical team had not allocated to shielding, evaluation revealed the following three themes: (1) genuine false positives due to how medications or comorbidities were text-mined by the algorithm (n=3, 7%); (2) no text-mining reason (n=5, 11%), which may indicate clinical judgement over-riding the national guidance; (3) clinical interpretation of national guidance (n=35, 81%). Further details and examples where possible are provided below. Of the three genuine false positives, two patients had issues regarding the temporality of the medication extraction. For instance, one letter containing the immunosuppressant medication in its ‘current’ section was followed by the reason for stopping in the future, which was still extracted as an active medication contributing to the shielding score. In one patient’s records, the mention of ‘pneumonia’ in the diagnosis section (that may be considered inactive/or an acute episode by the clinician) was mapped to pneumonitis as per the SNOMED dictionary, pneumonitis being a ‘parent code’, with pneumonia being one of the children of this term. According to the BSR guidance, any pre-existing lung disease (such as ‘pneumonitis’) would score a point.

The majority of false positives were due to the interpretation of the sentence in both sets of BSR guidance: ‘Patients who have rheumatoid arthritis (RA) or connective tissue disease-related interstitial lung disease (ILD) are at additional risk and may need to be placed in the shielding category.’ This sentence may have been interpreted by the clinical team as patients with RA alone or those with RA-ILD. The developed algorithm scored an additional 1 point for having RA, thereby tipping them into the shielding category. When RA was removed as scoring a point in the algorithm, the algorithm performance dropped considerably.

The 47 false negatives were also evaluated (ie, those patients who were deemed as requiring shielding but not detected as such by the algorithm). The primary reason for false negatives was medication metadata, specifically prednisolone dose not being correctly captured and incorporated towards the shielding score (n=28, 60%). This was often due to more complex instructions to the primary care team such as ‘Prednisolone 30 mg to be reduced in 5 mg increments every x days until reduced to a dose of 10 mg*’*. The algorithm captured the medication name correctly but was unable to calculate the current dose at the time of the pandemic, hence affecting the total score. No text-mining reason accounted for 25% (n=12) of false negatives. This included instances of patients being on a combination of two biological drugs for very active psoriatic arthritis and psoriasis, which would score a maximum of 2 points according to the guidance (3 required for shielding). Other examples included patients who had been receiving multiple intramuscular steroids, despite being on immunosuppressants for active/uncontrolled inflammatory arthritis, who may also be deemed clinically to be at much higher risk. In four individual patients (8.5%), there were extraction issues such as typographical errors contributing to misclassification. Examples include the prednisolone dose not extracted from the letters; hence, the patient scored 1 point rather than 2 as per the algorithm. Finally, clinical interpretation of the guideline accounted for 6% (n=3) false negatives. This primarily included patients with RA and ILD, where the decision for shielding as per the guidance was left up to the clinician (as above).

### Deployment of the algorithm and processing time

There were an additional 15 865 patients’ outpatient letters that had two letters available prior to April 2020 (including those that may no longer be active patients in the department). Table 4 shows the results of deploying the algorithm on these additional patients, which identified between 729 and 848 patients who met criteria for shielding using the two published BSR criteria. In terms of processing time, extracting diagnoses and medications (natural language processing (NLP) step) from free-text data took 18 hours for the 15 865 patients. However, once medications were extracted, running the shielding algorithm took an hour.

**Table 4:** Deployment of algorithm on whole patient cohort excluding training dataset (n=15 865)

	BSR scoring grid (numerical)	BSR risk stratification guide (non-numerical)
Patients advised to socially distance (%)	14 169 (89)	13 544 (85)
Patients advised to self-isolate/socially distance as per patient discretion (%)	967 (6)	1473 (9)
Patients advised to shield (%)	729 (5)	848 (5)

BSR, British Society for Rheumatology.

## Discussion

We successfully developed an automated text-mining algorithm to identify patients who required shielding as per national guidance based on patient demographics, medications and diagnoses. The automated algorithm demonstrated good specificity and reasonable sensitivity when compared with manual clinical decisions. Evaluation revealed the algorithm predominantly performed correctly against the rules. An automated algorithm for risk stratification has several advantages including reducing clinician time for manual review to allow more time for direct care, rapid deployment of a complex set of rules on all patient records across the whole department (rather than a subset) and improving efficiency and transparently communicating decisions based on individual risk. With further development, it has the potential to be adapted in other specialties that used similar risk stratification^12 13^ and for future public health initiatives that require prompt automated review of hospital outpatient letters.

When run across 15 865 patients, the data extraction and the developed algorithm deployment took 19 hours. If it is assumed that manual review for each patient, which requires review of multiple letters, takes on average between 7 and 10 min per patient, clinicians would be required to spend a total of between 104 and 149 hours reviewing a high-risk subset of 895 patients. Reviewing all 15 865 patients would have taken between 1850 and 2644 hours (77–110 days), which was simply not feasible given the need for prompt communication with patients in this setting. A recent survey in 2022, providing insights from nearly 1000 healthcare professionals, revealed that the average time spent generating clinical documentation was 13.5 hours a week.^14^ Consultant doctors spent on average 4.7 hours outside of normal working hours on such tasks. Additionally, the value of time for a consultant creating/adding to clinical documentation and searching for missing information was nearly £57 000 per doctor per annum.^14^ Automation of shielding guidance or future public health guidance requiring outpatient EHRs has several additional benefits, including cost implications of person-time. Importantly, it also allows for improving transparency of decisions, not only for patients deemed CEV but also those who were deemed to be of intermediate or low risk.

While we used clinical manual scores as the gold standard for assessing algorithm performance as it was the best available, physicians used their clinical discretion in some cases that over-rode the scoring system. An example may include an off-license but clinically appropriate use of two biologics in the same patient, which would score 2 according to the national rules, but a clinician may decide that this patient would be at high risk of infection therefore appropriately allocate them to the shielding category. Another key reason for lower sensitivity of 80% and false negatives from the evaluation was due to limitations in accurately assessing medication use during the risk window. This is a challenging area that needs to incorporate a range of complex tasks, including identifying the following: (1) the heading under which a given medication was listed; (2) any additional medication start/stop information that can over-ride the heading (eg, a medication listed under ‘current medications’ but with a note that the patient stopped taking it due to an adverse event but plans to restart at a later date); (3) the dose of drug in April 2020, particularly if there are plans to taper, pause or escalate, as was frequently the case with glucocorticoids; (4) duration of medication required to score specific drugs as per the national rules may not be always stated in the letter. For instance, the rule ‘intravenous cyclophosphamide within the last 6 months*’* requires a date of the last cyclophosphamide infusion within the letter that is clearly dated to calculate if it was administered within 6 months of 1 April 2020. To address these challenges, we combined a set of available tools (figure 3) to extract relevant information. However, the evaluation highlights the need for further refinement to improve identification and profiling of medications and diagnoses as reported in outpatient letters.

One way of addressing this would be to allow the algorithm to have a third result, apart from the advice to shield or not to shield. This could be using a rule-based approach to identify and single out expressions that contain complex medication instructions, such as multiple dosage mentions and temporal elements associated with a single medication name, to output a third category ‘for manual review’. Those records could be assessed individually by the medical team. Another solution would be to decide between the clinical team a set of a priori additional ‘rules’ that would allow the algorithm to increase the score when certain clinical exceptions were encountered (e.g., being on two biological drugs).

While the algorithm performed correctly against the set of rules, in the case of 11% of false positives and 25% of false negatives were not due to failures in the automated extraction and algorithm. Evaluation of these instances implied that clinical judgement correctly over-rode the national set of rules, as discussed above. The later published BSR risk stratification guide (online supplemental figure 2) also included assessment of disease activity with rules such as ‘well-controlled patients with minimal disease activity*’*. However, disease activity measures are not always captured in the semi-structured parts of letters and may be recorded in the free-text part of the letter. As opposed to clinicians, the algorithm did not have access to the narrative part of clinical outpatient letters, which might have further information on the current medication status, as well as information on disease severity used in the decision-making. Therefore, this rule was out of scope for the algorithm, however, could be introduced following future development.

From a patient perspective, qualitative research has described the increased emotional work of feeling forgotten about^15^ and navigating the conflicting advice given when notifications came from multiple different sources.^16^ Patients value timely, clear communication about their shielding status. Efficient deployment of the developed algorithm may have improved this aspect of the shielding process. From a clinician perspective, during the pandemic, rheumatology clinicians were also redeployed to help with the front line, which in some cases led to clinicians feeling there was less time to directly support rheumatology patients despite wanting to do so.^15^ Automating clinical processes that are administrative and time-consuming would have allowed more time for these interactions with rheumatology patients.

The results of the study need to be interpreted in the context of certain limitations. The first is in line with the information governance and approvals of the study; we did not access the patient records of the 64 patients who opted out as part of the national data opt-out scheme.^17^ Therefore, should such algorithms be implemented in real time in the future for clinical purposes, consideration needs to be made on how to handle decisions and communicate those clinical decisions. Second, we did not have access to the free-text information within letters due to the higher risk of patient re-identification. Therefore, information regarding disease activity, medication dose adjustments or other social factors that may have influenced the clinical decision to shield were not available. We have however used this work to leverage further funding to build on the existing tools.^18^ Within this programme, we plan to use a suite of deidentification software that will be applied to the free-text narrative of each letter to remove explicit mentions of key personal identifiable information. This would reduce the risk of accidental reidentification to researchers and enable further NLP development to extract other measures of interest such as disease activity and quality of life. Third, the process to develop the algorithm was an iterative one, which was not included in the above deployment time as not accurately captured across different team members. However, once developed, it would be able to be easily adapted for future public health risk stratification if needed. Since development of the algorithm that relied on rule-based NLP, large language models (LLMs) such as GPT4 have surged in popularity and are being leveraged in several settings. However, LLMs are associated with their own limitations including black box models, LLM hallucinations and introducing different types of bias.^19–22^ How they could be implemented in a healthcare setting on outpatient letters that contain sensitive patient information to replace text analytics has not been well established.

Beyond COVID-19, the algorithm has the future potential to be adapted for several purposes that include addressing questions regarding service provision, clinically important research and related to population health. For instance, it is currently not possible to easily determine the prevalence of a particular group of conditions such as inflammatory arthritis within a hospital, due to a lack of structured coded diagnoses in an outpatient setting. The developed algorithm could be adapted to address what proportion of rheumatology patients with a certain diagnosis are on a specific biological drug or medication. This type of information would be extremely useful for cohort selection to aid clinical trial recruitment, for quality improvement purposes and to estimate financial costs for local authorities with a specific condition/medications with consequent policy implications. Quickly identifying patients on specific medications within clinic letters could help provide a safety net for DMARD monitoring. Capturing dose and frequency of the medications across letters can enable insights into specific clinical/research questions such quantifying dose tapering across a department and subsequent cost-savings.

The COVID-19 pandemic has illustrated the pertinent public health need to efficiently use data collected at the point of care to rapidly evaluate comorbidities and medications timely and efficiently. Establishing partnerships between academia, secondary care business intelligence teams, patient and clinical representatives can allow development of new algorithms using text analytics that could ultimately improve patient care and save clinician time, in an already overstretched health service.

## Data Availability

The data used for the development of the algorithm are not publicly available without completion of specific training requirements and information governance checks mandated by the data owners. The shielding algorithm code will be made available for use on reasonable request.
