# Supplementary material for: Development and evaluation of a text analytics algorithm for automated application of national COVID-19 shielding criteria in rheumatology patients
Source: Ann Rheum Dis. 2024 Apr 4;83(8):1082–91. doi: 10.1136/ard-2024-225544 (PMC11287580; doi:10.1136/ard-2024-225544)
Supplement: Supplementary data [file ard-2024-225544supp001.pdf]

Supplementary material

Supplementary Figure 1: An example of an outpatient letter aligned to the Professional Record Standards Body Outpatient Letter Standard.

Date of appointment 3rd Jan 2018
Clinic: RHEUMATOLOGY
Date of Birth: 28/10/48

Rheumatological Diagnosis: Psoriatic arthritis with peripheral and axial involvement (confirmed on MRI)

Non-rheumatological Diagnosis: Psoriasis
Type 2 Diabetes

Medications: Adalimumab 40mg subcut fortnightly (Aug 2015)
Folic Acid 5mg weekly
Methotrexate 20mg weekly

Previous DMARDs: Cyclosporin 100mg daily

Assessment:
Mrs Smith was reviewed in rheumatology clinic with a flare of her psoriatic arthritis. She reported experience pain and swelling in her right knee for the last two days, which is affecting her mobility considerably....

Supplementary Figure 2: COVID-19 shielding grid (without scores)

British Society for Rheumatology

COVID-19 - Identifying patients for shielding in England

**Risk stratification guide**	Patients to shield	Patients to self-isolate or maintain social distance at their discretion	Patients to maintain social distance
Immunosuppressive medication	• Corticosteroid dose of ≥20mg (0.5mg/kg) prednisolone (or equivalent) per day for more than four weeks<br>• Cyclophosphamide at any dose orally or within last six months IV<br>• Corticosteroid dose of ≥5mg prednisolone (or equivalent) per day for more than four weeks plus at least one other immunosuppressive medication*, biologic/ monoclonal** or small molecule immunosuppressant (e.g. JAK inhibitors)***<br>• Any two agents among immunosuppressive medications, biologics/monoclonals** or small molecule immunosuppressants with any co-morbidity****	• Well-controlled patients with minimal disease activity and no co-morbidities on single agent broad spectrum immunosuppressive medication, biologic/monoclonal** or small molecule immunosuppressant<br>• Well-controlled patients with minimal disease activity and no co-morbidities on single agent broad spectrum immunosuppressive medication plus Sulphasalazine and/ or hydroxychloroquine<br>• Well-controlled patients with minimal disease activity and no co-morbidities on a single agent broad spectrum immunosuppressive medication* at standard dose (e.g. Methotrexate up to 25mg per week) ***plus*** single biologic (eg anti-TNF or JAKi)** or ***	• Single agent 5-ASA medications (eg mesalazine)<br>• Single agent6-mercaptopurine<br>• Only inhaled or rectally administered immunosuppressant medication<br>• Hydroxychloroquine<br>• Sulphasalazine

* Immunosuppressive medications include: Azathioprine, Leflunomide, methotrexate, Mycophenolate (mycophenolate mofetil or mycophenolic acid), ciclosporin, cyclophosphamide, tacrolimus, sirolimus. It does **NOT** include Hydroxychloroquine or Sulphasalazine either alone or in combination.

** Biologic/monocolonal includes: Rituximab within last 12 months; all anti-TNF drugs (etanercept, adalimumab, infliximab, golimumab, certolizumab and biosimilar variants of all of these); Tociluzimab; Abatacept; Belimumab; Anakinra; Seukinumab; Ixekizumab; Ustekinumab; Sarilumumab;

*** Small molecules includes: all JAK inhibitors – baracitinib, tofacitinib etc

**** Co-morbidity includes: age >70, Diabetes Mellitus, any pre-existing lung disease, renal impairment, any history of Ischaemic Heart Disease or hypertension. Patients who have rheumatoid arthritis (RA) or CTD-related interstitial lung disease (ILD) are at additional risk and may need to be placed in the shielding category. All patients with pulmonary hypertension are placed in the shielding category

NB This advice applies to adults, children and young people with rheumatic disease. We do **NOT** advise that patients increase steroid dose if they become unwell

V3. Published: 24 March 2020
